# Intersection between Obesity, Dietary Selenium, and Statin Therapy in Brazil

**DOI:** 10.3390/nu13062027

**Published:** 2021-06-12

**Authors:** Ligia M. Watanabe, Anderson M. Navarro, Lucia A. Seale

**Affiliations:** 1 Department of Health Sciences, Division of Nutrition and Metabolism, Ribeirão Preto Medical School, University of São Paulo—FMRP/USP, Ribeirão Preto 14040-900, SP, Brazil; ligia_watanabe@usp.br (L.M.W.); navarro@fmrp.usp.br (A.M.N.); 2 Pacific Biosciences Research Center, School of Ocean and Earth Science and Technology, University of Hawaii at Manoa, Honolulu, HI 96822, USA

**Keywords:** obesity, selenium, Brazil, statins

## Abstract

Obesity is among the most alarming health concerns, impacting public health and causing a socioeconomic challenge, especially in developing countries like Brazil, where approximately one quart of the population presents obesity. As an established risk factor for numerous comorbidities with a multifactorial etiology, obesity is a consequence of energy-dense overfeeding, however with significant undernourishment, leading to excessive adipose tissue accumulation and dysfunction, dyslipidemia, and micronutrient deficiencies. About 60% of patients with obesity take statins, a cholesterol-lowering medication, to curb dyslipidemia, with ~10% of these patients presenting various myopathies as side effects. Statins act upon the rate-limiting enzyme of cholesterol biosynthesis in the liver, which is a pathway providing intermediates to the synthesis of selenoproteins, i.e., enzymes containing the micronutrient selenium. Statins have been postulated to negatively impact selenoprotein synthesis, particularly in conditions of selenium deficiency, and potentially implicated in the myopathies occurring as side effects of statins. The Brazilian population is prone to selenium deficiency, hence could be considered more susceptible to statin side effects. This review examines the specific consequences to the Brazilian population of the harmful intersection between obesity development and concomitant micronutrient deficiencies, particularly selenium, combined with statin treatment in the context of nutrition in Brazil.

## 1. Introduction 

The increasing prevalence of obesity has become a worldwide health problem and a significant challenge to chronic disease prevention and health across the life course [1,2,3,4]. The World Health Organization (WHO) estimated that globally approximately 1.9 billion adults aged 18 years and older were overweight in 2016. Of these, over 650 million adults had obesity [5]. In Brazil, according to the National Survey of Health, the percentage of adults with obesity more than doubled in 17 years, changed from 12.2% (2002–2003) to 26.8% (2019) [6]. At the same period, the proportion of overweight adults changed from 43.3% to 61.7% and represented almost two-thirds of the Brazilian population [6]. In the group aged 18 and over, 25.9% had obesity in 2019, a total of 41.2 million people [6].

Obesity is among the most alarming health concerns, impacting public health and causing a socioeconomic challenge [7]. The economic costs of obesity have raised considerable attention in recent years, with an existing gradient between increasing body mass index (BMI) and costs attributable to obesity [7], and this concern may be amplified in developing countries such as Brazil [8]. Studies focusing on the economic burden of obesity in Brazil revealed the estimated costs of diseases connected to overweight and obesity occurrence reached almost US$2.1 billion in one year, with 68.4% of total costs due to hospitalizations [7,8]. A different study calculated direct costs attributable to obesity in Brazil as US$269.6 million and morbid obesity as US$64.2 million [7,9]. Using microsimulation, healthcare costs were predicted to increase from $5.8 billion in 2010 to $10.1 billion in 2050 [7,10]. Besides the financial impact of obesity and its comorbidities on the Brazilian health system, obesity also imposes costs in loss of productivity and quality of life to society and individuals [7,8,11].

Modern obesity is a pathology and an established risk factor for other noncommunicable diseases (NCDs), including type 2 diabetes, cardiovascular disease, dyslipidemia, depression, hypertension, cancer [11,12,13,14], while also being a risk factor for the development of severe COVID-19, a communicable disease [15]. According to WHO, NCDs were responsible for 71% of all deaths worldwide and 77% of all NCD deaths are in low- and middle-income countries, like Brazil [13,16]. Obesity has a multifactorial etiology [11,17], resulting from a complex interaction between genetic, environmental, social, and behavioral factors [11,18,19,20,21]. Usually, a consequence of energy-dense nutrition and reduced energy expenditure [20,22], obesity is commonly associated with macronutrient metabolism impairment, particularly lipid and carbohydrate; however, micronutrients can also impact the development of obesity and impair energy metabolism [20,21]. 

The percentage of patients with obesity using cholesterol-lowering medications is about 60%, with statins being the most widely prescribed medication [23]. Statins or 3-hydroxy-3-methylglutaryl coenzyme A (HMG-CoA) reductase inhibitors, are the most effective pharmacological therapy treatment for hypercholesterolemia and cardiovascular events prevention [24,25,26]. HMG-CoA reductase is the rate-limiting enzyme of cholesterol biosynthesis, catalyzing HMG-CoA reduction to mevalonic acid, and the main target of statins [27]. 

Notably, the cholesterol biosynthesis pathway is also important to the metabolism of selenium, an essential micronutrient present as a critical residue of multifunctional selenoproteins [28,29], as a downstream intermediate is isopentenyl pyrophosphate, necessary for the selenocysteine tRNA (Sec tRNA) maturation and proper selenoprotein synthesis. When cholesterol biosynthesis is inhibited at the HMG-CoA reductase step, diminished availability of isopentenyl pyrophosphate and consequently, of selenoproteins, may occur [30,31]. Thus, selenoprotein deficiency could be associated with statin use, and this deficiency is chiefly worrisome in tissues where selenoproteins are involved in controlling energy metabolism, such as skeletal muscle [30,31,32,33,34]. Moreover, deficiency of selenium and metabolic impairment in selenoprotein synthesis could occur in individuals with obesity and may aggravate side effects of statin therapy. Insufficient selenium intake [35,36] and lower blood concentration of this micronutrient [37,38] have been reported in several areas of Brazil, mainly due to the lower selenium content in most Brazilian soils used for agriculture [39].

This review examines the specific nutritional consequences to the Brazilian population of the harmful intersection between obesity development and concomitant micronutrient deficiencies, particularly selenium, and its implications in statin therapy. 

## 2. Obesity: Overfed but Undernourished

Obesity is known to have a multifactorial etiology [11,17], resulting from a complex interaction between genetic, environmental, social, and behavioral factors [11,18,19,20,21]. Gene-environment interactions are generally accepted as primary cause of obesity [18]. Several attempts using genome-wide association studies (GWAS) have been performed to identify obesity gene variants responsible for enhanced susceptibility, with approximately 140 obesity susceptibility genes found to be associated with adiposity measures such as BMI, body fat percentage, and waist circumference [18]. Nevertheless, the “obesogenic environment” includes additional factors such as dietary nutrients, age, gender, ethnicity, duration of sleep, amount of physical activity, sedentary behavior, stress, smoking, alcohol consumption, use of medication, and depression [40,41,42,43], not all of them responding singularly to genotypical differences.

### 2.1. Nutritional Aspects of the Obesogenic Environment in Brazil

An obesogenic environment includes all external aspects linked to possible cause-effects of generating obesity. Reduced intake of fruit, vegetables, nuts and seeds, whole grains, and fish; and elevated intake of red or processed meat, potato chips, sugar-sweetened drinks, sodium, saturated trans-fat, and added sugar are considered risk factors to obesity development [44]. Recently, authoritative reports recognized ultra-processed foods (UPF) as definers of unhealthy diets. UPFs are ready-to-eat foods constituted entirely or predominantly from substances extracted from food such as oils, fats, sugar, proteins, or derived from food constituents, such as hydrogenated fats, modified starches, or yet synthesized from organic materials such as dyes, flavorings, flavor enhancers, and other additives used to alter the food’s sensory properties [45,46,47,48,49,50,51]. 

A Brazilian study of national trends over 25 years on household food acquisition and health implications concluded that diets with high proportions of UPFs are unbalanced and damaging to health, supporting the association of UFPs intake with obesity and related cardiometabolic outcomes [45].

### 2.2. Dietary Patterns of the Brazilian Population

Comparison between the nutrient profile of two purchase shares, one composed exclusively of UPFs and the other restricted to non-UPFs, in total household food purchases in Brazil and Chile showed that the UPF share (or the “average” UPF) was significantly higher in non-communicable diseases (NCD) promoting nutrients (free/added sugars, sodium) and energy density, and lower in NCD-protective nutrients (protein and fiber) than the non-UPF share. In Brazil, the “average” UPF presented higher content of NCD-promoting saturated fat [45,46,47,48,49]. The results highlighted an observed trend in the Brazilian food pattern: the substitution of traditional meals, rich in natural or minimally processed foods, to UPFs. Recommendation to avoid consumption of UPFs is also supported by these studies [45,46,47].

It is difficult for the human body to regulate energy balance with high-energy-density diets. Both excessive free sugar or saturated and trans-fat increase the risk of excessive weight gain and obesity and affect morbidity and mortality from cardiovascular diseases. Moreover, insufficient fiber intake increases the risk of obesity, type 2 diabetes, cardiovascular diseases, and several types of cancer, while reduced potassium intake increases high blood pressure [48,49,50,51]. These studies provide additional evidence that the dietary share of UPFs determines the nutritional quality of diets, imprinting a universal significance, particularly in countries where rates of obesity and other diet-related chronic non-communicable diseases continue to increase rapidly. At the same time, the prevalence of micronutrient deficiencies has persisted.

### 2.3. Nutrient Deficiency

An adequate, healthy diet should be accessible physically and financially, balanced in quantity and quality, and satisfying variety, moderation, and pleasure. Moreover, it should arise from practices of production and distribution that are sustainable [52]. There are interactions among isolated nutrients, and people consume these interactions in food as a whole. Hence, the adequacy of one nutrient does not necessarily reflect a healthy dietary pattern. The promotion of an adequate and healthy diet encompasses strategies aiming to provide personal and social ways to eat well in biological, social, cultural, economic, and political aspects while considering the sustainability of natural resource usage and environmental protection [53]. Thus, the high energy intake evidenced in obesity has no direct relationship with the nutritional adequacy of nutrients.

Obesity is usually a consequence of overeating; however, it can paradoxically be associated with deficiencies in nutrient intake and status. The prevalence of nutritional deficiencies among individuals with obesity has been established to be higher than in people of the same age and sex with normal weight [54]. The identification and isolation of nutrients present in foods and the understanding of the effects of these individual nutrients on the incidence of specific diseases are at the core of modern nutritional sciences [45,46,47]. Regarding obesity, a significant number of patients presented an effect of the “obesogenic diet,” i.e., dense in energy but devoid of several nutrients. An obesogenic diet is unbalanced, containing highly processed fast foods, rich in carbohydrates with added sugar, saturated fat, and sodium, and often deficient in several vitamins and micronutrients, such as retinol, ß-carotene, vitamin D, vitamin E, vitamin C, folate, iron, calcium, and selenium [54]. 

The increase in obesity in recent decades is associated with changes in the population’s ways of life, including wide-ranging dietary changes, especially in micronutrient intake [55]. Micronutrients are essential to several aspects of human metabolism, being necessary for the development and health of individuals. There is no global assessment of the nutritional status of the micronutrients in Brazil, mainly in obesity condition, but studies pointed to the necessity to monitor dietary trends that could lead to deficiencies with adverse consequences for the population health [55].

## 3. Selenium and Selenoproteins

The biological activity of selenium is mainly carried out by selenoproteins, a small class of 25 proteins that contain the amino acid selenocysteine (Sec). This amino acid is incorporated into selenoproteins after translational re-coding of the UGA codon, commonly signaling a stop, to Sec, a process requiring several *cis* and *trans* factors, such as a dedicated elongation factor and the Sec-insertion sequence binding protein 2 (SECISBP2). This molecular mechanism has been thoroughly reviewed elsewhere [56,57,58,59,60,61,62]. Sec can be directly acquired through the diet or recycled by the actions of the enzyme Sec lyase (SCLY) [63]. However, to be utilized in selenoprotein synthesis, Sec needs to be resynthesized in its Sec tRNA. The Sec tRNA is primarily loaded with a serine moiety. The conversion of serine into Sec occurs by the concerted actions of two enzymes, selenophosphate synthetase 2 (SEPHS2) and O-phosphoseryl-tRNASec kinase (PSTK) [64] in the presence of selenophosphate, produced from selenide. All selenium forms in eukaryotic cells have to be converted into selenide to be used in the mechanism of selenoprotein synthesis.

### 3.1. Disruptions in Selenoprotein Synthesis

Impairment in the levels or activity of either participants of the selenoprotein synthesis machinery or specific selenoproteins affects health. Established roles of specific selenoproteins in thyroid hormone activation, male reproduction, immune responses, neuronal development and activity, and redox homeostasis corroborate the essentiality of this micronutrient in health. Selenoprotein synthesis factors’ actions on specific physiological processes in various tissues have been demonstrated in depth. Significant advances were achieved using mouse models either lacking or with mutations in the Sec tRNA gene, *Trsp*, or studies of mutations in the *SECISBP2* gene in humans. Whole-body removal of *Trsp* is embryonically lethal, while targeted deletion of *Trsp* in specific mouse tissues led to differing pathological features, such as myocardial failure, hepatocellular necrosis, cerebellar hypoplasia, and premature death, and mutations affecting prominently the expression of a specific subset of stress-related selenoproteins (reviewed in [65]). On the other hand, people with rare mutations of the *SECISBP2* gene leading to SECISBP2 deficiency developed growth retardation with thyroid hormone abnormalities, including a 12-year-old Brazilian girl with a nonsense mutation that also presented a strong obesity phenotype [66]. Thyroid abnormalities due to *SECISBP2* mutations compromised the actions of selenoproteins type 2 deiodinase (DIO2), plasmatic selenoprotein P (SELENOP), and antioxidant enzyme glutathione peroxidase (GPX) [67], partially explaining the disorders presented by patients. Moreover, mice lacking *Scly*, which encodes the Sec-decomposing enzyme SCLY that participates in selenoprotein synthesis and degradation, develop obesity with impaired hepatic energy metabolism and redox status, and localized selenium deficiency [68,69,70]. When mice lacking *Scly* were treated with statin, the response to medication were strongly sex-dependent, with selected regulation of the expression of selenoproteins in the liver and skeletal muscle, particularly the genes for *Selenon*, *Gpx1,* and *Selenop* [71].

### 3.2. Selenium Intake and Status of the Brazilian Population

Selenium is a dietary micronutrient essential to humans, and its levels in food usually reflect the content in the surrounding environment. Brazil’s large territorial area warrants a great potential diversity in the content of selenium in soils. Hence, plants and livestock may present varying selenium amounts, according to the local soil characteristics where they are growing. As a general pattern, northern areas of the country present higher selenium content in the soil than the southern areas [72]. Seleniferous soils are prevalent in the Amazon region, where native plants such as Brazil nuts (*Bertholletia excelsa*) grow. Brazil nuts are the richest dietary source of selenium and considered a useful food source to improve selenium status [73], particularly in populations where selenium deficiency prevails. Nevertheless, the selenium concentration of Brazil nuts is highly variable according to the area where growth and harvesting occur, and food processing mechanisms utilized in commercial enterprises [74]. Besides Brazil nuts, additional valuable sources of selenium in Brazilian diets include seafood-related items such as tuna and sardines, meat and poultry, egg yolks, and wheat flour items [36].

The recommended daily allowance (RDA) of selenium for adult humans is 55 µg, as suggested by the Food and Agriculture Organization (FAO) of the United Nations. Nevertheless, guidelines are country-specific, established by nutritional regulatory bodies that consider selenium availability in fertile soils and livestock, dietary preferences, and supplementation intake. Both United States and Brazilian nutritional guidelines for selenium follow FAO guidelines and recommend a daily intake of selenium to be 55 µg [75], which safeguards selenoprotein synthesis and, consequentially, adequate health. Notably, selenium deficiency is rare in the United States, with most American populations obtaining at least RDA levels of selenium in their diets [76]. On the other hand, selenium deficiency is more commonly found in Brazil, possibly due to food sources from predominant selenium-deficient soils in top agricultural regions and socio-economical patterns affecting food choices. Moreover, selenium supplementation in specific areas may play a role in this variation, particularly livestock supplementation and food processing. In Brazil, processed products derived from wheat, such as bread, may contain selenium-supplemented yeast and are significant sources of dietary selenium for the Brazilian population [77].

## 4. Intersection between Dietary Selenium and Obesity in Brazil

Attempts to obtain an association between selenium intake or status and obesity (considered as BMI ≥ 30) have been peppered by variations according to the studied population, with regional food sourcing differences, gender, pathological state, individual genotype, and socioeconomic criteria significantly weighing in the final association analysis (summarized in Table 1). 

A systematic review of observational studies analyzing antioxidant intake and obesity found no directional association between selenium intake or status and obesity [78]. On the other hand, lower serum selenium has been connected with overweight and obesity, both in men and premenopausal women in the USA [79] and France [80]. Conversely, high serum selenium has also been associated with obesity prevalence, particularly in hypertension individuals from Russia [81]. Nevertheless, despite these conflicting findings, the variabilities listed above warrant more granular perspectives, and this section focuses on the association of selenium and obesity in the Brazilian population. 

A cross-sectional study of premenopausal women from a northeastern area of Brazil, where seleniferous soils prevail, found that plasma selenium concentration was negatively associated with obesity and visceral adiposity. The study also unveiled that women with obesity had elevated selenium excretion, suggesting a causal relationship to be further explored [82]. The same research group reported results from a different study within the same geographical area corroborating the negative association between plasma selenium and obesity, with higher clearance through excretion [83] but not higher selenium levels in the urine. Interestingly, the latter study also unveiled a negative relationship between selenium and free thyroxine (T4) and 3-3’-5-triiodo-L-thyronine (T3), both thyroid hormones regulating energy metabolism and controlled by the hypothalamic-pituitary-thyroid axis; however, the observed increases in thyroid hormone in women with obesity were not sufficient to indicate compromised thyroid function. 

Alarmingly, a cross-sectional survey of 2–11 months old infants also from the selenium-rich northeastern region of Brazil revealed 91% of subjects to present selenium deficiency [84], a finding that may increase the risk for future obesity development in the population. As other micronutrient deficiencies were also present in these infants, it is suggested that these deficiencies reflect the predominant low-income setting in which the study was conducted, which leads to inadequate nutritional practices favoring empty calories from processed foods. Such deficiencies were particularly worrisome among overweight infants, where micronutrient deficiencies and overweight/obesity can generate a “double burden malnutrition,” compromising future health. 

A large study conducted with Brazilian adolescents (12–17 years of age) from all areas of the country showed the prevalence of selenium adequacy in terms of intake, with most selenium being acquired from meat, poultry, and products derived from wheat [85]. Intake of UPFs was correlated with inadequate selenium intake in adolescents from the northeastern region of Brazil [86]. As overconsumption of UPFs increases the risk of obesity development [87], it is tempting to suggest a causal relationship involving overconsumption of UPF, leading to obesity, and consequently low selenium levels, and this possibility warrants further research. Interestingly, an observational study in adolescents with severe obesity from Rio de Janeiro identified 36% of subjects as selenium-deficient based on serum selenium levels, and the low proportion of deficiency may be related either to the small sample size of the study or specific food sources of selenium prevailing in this study [88]. Comparatively, a small study of healthy adult volunteers assessed for selenium levels in the same city presented mild selenium deficiency, particularly among men. Despite a prevalence of overweight and obesity among participants, unfortunately, no association between selenium and obesity was attempted in this study [89].

## 5. Statins and Associated Side Effects: Occurrences in the Brazilian Population

### 5.1. Lipid Dysregulation in Obesity

Obesity is a condition associated with intensive remodeling of adipose tissue, changes in adipose tissue composition with altered adipokines secretion, and development of adipose tissue dysfunction (adiposopathy) [18], adversely affecting nearly all functions of the body [7].

The typical dyslipidemia of obesity consists of increased triglycerides and free fatty acids, decreased high-density lipoprotein cholesterol (HDL), and normal or slightly increased low-density lipoprotein cholesterol (LDL). The concentrations of plasma apolipoprotein (apo) B are also often increased, partly due to the hepatic overproduction of apo B-containing lipoproteins [22,90,91]. People with hyperlipidemia have a higher risk of developing cardiovascular disease when compared to people with normal total cholesterol levels [91]. Population-based observational studies conducted in the Brazilian population indicated the prevalence of dyslipidemia to be 43% to 60% in adults [92,93]. Mortality rate from cardiovascular disease was 359,000 in 2017, representing 27.3% of all deaths [94]. Considering the importance of controlling cholesterol levels in the bloodstream, inhibiting cholesterol biosynthesis becomes essential [90]. 

**Figure 1:**
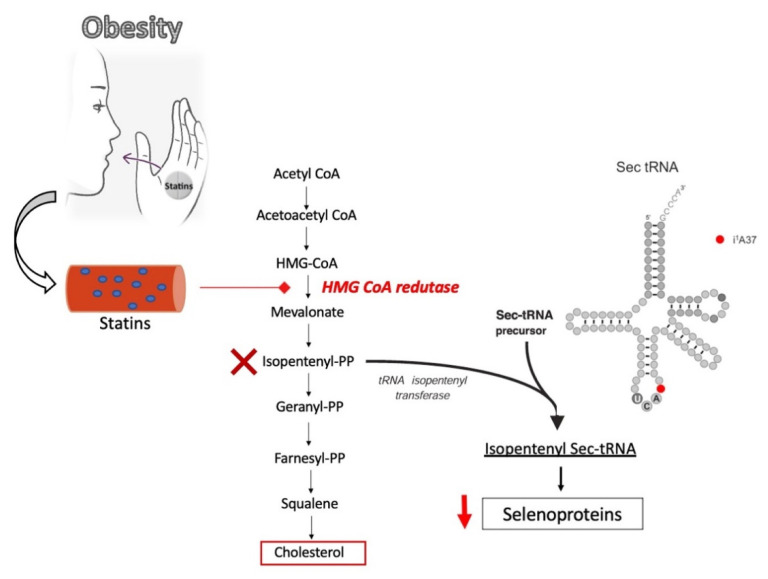
Interconnection between the cholesterol biosynthesis pathway, statins mode of inhibition of the HMG-CoA reductase step, and its consequential reduction in downstream products such as isopentenyl pyrophosphate (PP), which is necessary for the maturation of the tRNA for selenocysteine (Sec-tRNA).

The cholesterol biosynthetic pathway is regulated at several points [90,95], with some intermediaries being diverted and either used as precursors in the biosynthesis of other compounds or to perform specific functions on the body (Figure 1) [90]. Generally, this pathway can be categorized into two stages: (a) Condensation of isoprenoid units yielding squalene; and (b) cyclization of squalene to produce lanosterol, which is subsequentially converted to cholesterol [95]. Biosynthesis of cholesterol starts with the formation of 3-hydroxy-3-methylglutaryl-coenzyme A (HMG-CoA) from one molecule of acetyl-CoA and one of acetoacetyl-CoA. HMG-CoA is then converted to mevalonate by the action of HMG-CoA reductase (HMGCR). This reaction is often considered the rate-limiting step in cholesterol biosynthesis and serves as an essential target of cholesterol-lowering drug statin [95]. 

### 5.2. Statin Use in Brazil

Statins are inhibitors of the hepatic HMGCR enzyme and are the most prescribed and effective pharmacological therapy for hypercholesterolemia and the prevention of cardiovascular events [24,25,26]. The percentage of patients with obesity using cholesterol-lowering medications is about 60%, with statins being the most widely prescribed medication [23]. Approximately 25% of the world population above 65 years-old takes a statin for primary or secondary prevention of cardiovascular diseases [96].

Statins became available within the Brazilian healthcare system in 2002. Currently, five statins are provided by the Brazilian Unified Health System (SUS): atorvastatin, fluvastatin, lovastatin, pravastatin, and simvastatin [97]. Simvastatin is the most cholesterol-lowering statin prescribed in the Brazilian SUS [98], followed by atorvastatin and rosuvastatin [97]. 

### 5.3. Statin-Associated Side Effects among Brazilians

**Figure 2:**
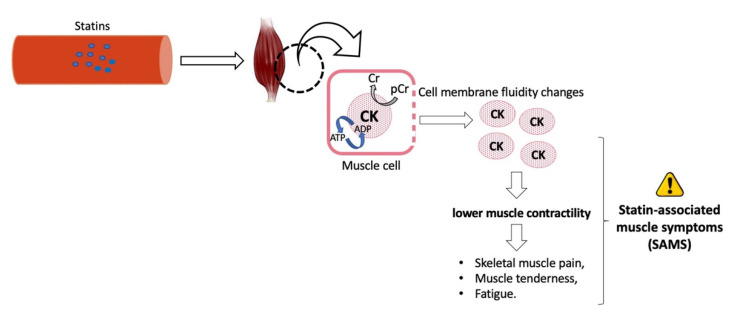
Development of statin-associated muscle symptoms (SAMS), in which the cells of the skeletal muscle enhance its cycling of creatine (Cr)/phosphocreatine (pCr) and its cell membrane permeability, facilitating the release of creatine kinase (CK) to the circulation.

Despite the favorable safety profile of statins [24,99,100], these pharmacological drugs still evoke distinct side effects with unclear molecular origins [30]. Statin-associated muscle symptoms (SAMS) are the most commonly reported adverse effect of statins (Figure 2). About 10% to 29% of patients receiving statin therapy present SAMS in the clinical practice and according to observational studies, respectively [25,100,101]. In Brazil, the percentage of patients that are intolerant to statins varies between 5 and 10%. Adverse muscular events related to statin treatment were found in 17% of patients treated in the University Hospital in Sao Paulo city, Brazil [102]. 

The definition of SAMS often varies according to different guidelines [101]. Definitions range from myalgia to elevation of creatine kinase (CK) to clinical rhabdomyolysis [25,100]. CK is an enzyme found in both cytosol and mitochondria of tissues where energy demands are high, most notably skeletal muscle. Elevated serum CK activity can indicate muscular tissue damage and are observed in several pathological conditions, including SAMS (Figure 2) [25,103,104]. Several factors can increase SAMS risk, including high doses of statin, hypothyroidism, the interaction of statins with other drugs that inhibit statin catabolism, reduced muscle mass, and increased physical activity. Additionally, advanced age, being female, and alcohol consumption have also been shown to increase SAMS risk [25,101]. SAMS in Brazil has been negatively associated with age, educational level, the residential area, polypharmacy, and alcohol consumption [97].

Several hypotheses have been proposed to elucidate the mechanisms of SAMS development [26]. One hypothesis suggests a possible mitochondrial dysfunction and cellular energy utilization connected to the depletion of coenzyme Q10 in muscle. Additional suggested mechanisms for SAMS development include increased oxidative stress in muscle cells, decreased intracellular concentrations of cholesterol, impairment of calcium homeostasis, immune response, and aberrant expression of ryanodine receptors (RyR), and reduction of downstream intermediaries of the mevalonate pathway [100,105,106].

Notably, isopentenyl pyrophosphate, an intermediate of the mevalonate pathway, is responsible for the isopentenylation of adenosine at position 37 in tRNAs that generates isopentenyl adenosine (i6A) (Figure 1). Interestingly, i6A is necessary for effective translation of Sec codons by the Sec tRNA [30,31,32,33,34,107]. When isopentenyl pyrophosphate levels are low, as when statins are used, reduced synthesis of selenoproteins may occur [30,31]. Selenoprotein deficiency has been associated with the prevalence of side effects of statins, such as myopathies and increased oxidative stress [98]. Reduced expression of selenoprotein N (SELENON) and GPX have been pointed as potentially involved in statin-induced myopathy, due to their participation in mechanisms that maintain muscle and cellular redox homeostasis [98], but it is notable that selenium-supplemented mice treated with statins showed unaltered protein levels of SelenoN [71].

### 5.4. Selenium Supplementation and SAMS in Brazil

As the prevalence of an inverse association between selenium and obesity was unveiled in different populations throughout Brazil, intervention attempts to correct selenium deficiency in individuals with obesity were pursued. Most interventional studies supplement selenium using Brazil nuts as a selenium source, which predominantly has the highly bioavailable amino acid selenomethionine. Brazil nut supplementation for 16 weeks improved the lipid profile of female adolescents from Rio de Janeiro and with obesity [108]. A different study enrolling women with severe obesity and supplemented with an 8-week regimen of Brazil nuts also improved their lipid profile and plasmatic GPX activity [109]. These studies suggest that correcting selenium deficiency by introducing Brazil nuts in a diet may help curb some of the lipid dysregulation observed in obesity which can have extended effects in avoiding SAMS. Interestingly, there were benefits of the consumption of one Brazil nut/day for three months on the selenium status, oxidative stress, body weight, and muscle parameters by patients using statins [98]. Additionally, a single nucleotide variation in the gene for selenoprotein P was associated with antioxidant, muscular, and lipid biomarkers in response to Brazil nut consumption by patients using statins, highlighting the role of individual variation in the metabolic response to selenium intake [110]. These approaches for selenium supplementation substantiate the relevance of Brazil nut on assisting in health outcomes and support the promotion of a local product originating from the Brazilian Amazon to improve selenium deficiencies derived from obesity in the Brazilian population. 

## 6. Final Considerations

This review provided insights into the magnitude of obesity in the Brazilian population, the influence of obesity in selenium deficiency, the role of the environment as a determinant factor for selenium deficiency, and how the combination of these factors could increase the risk of muscle symptoms derived from statin use. There is potentially a promising role for selenium supplementation in Brazilian patients with obesity, especially for those using statins, using food items available to the Brazilian population, such as Brazil nuts. Nevertheless, considering the variations in selenium recommendation, including individual and geographical characteristics and food habits, it is crucial to optimize selenium intake on a personalized, precise scale, considering the quantity and the selenium chemical form to assure the efficacy and safety of selenium supplementation. 

## Figures and Tables

**Table 1:** Summary of discussed studies associating dietary selenium and obesity.

Reference	Study Population	Country	Conclusion
Galan et al., 2005 in Hosseini et al., 2017 (review) [78]	3128 adults (58 % women)	French	No association between zinc and selenium concentration and anthropometric data
Tascilar et al., 2011 in Hosseini et al., 2017 (review) [78]	34 with obesity and 33 healthy children	Turkey	No differences in serum trace element levels (selenium and zinc) between subjects with obesity and healthy ones
Kimmons et al., 2006 [79]	16,181 adults	United States of America	Lower serum selenium was associated with overweight and obesity, both in men and premenopausal women
Arnaud et al., 2006 [80]	13,017 subjects (7876 women and 5141 men)	French	Obesity was associated with decreased serum selenium levels only in women
Tinkov et al., 2021 [81]	395 adults (199 lean and 196 with obesity)	Russia	High serum selenium was associated with obesity prevalence, particularly in hypertensive individuals
Soares de Oliveira et al., 2021 [82]	139 women (63 with obesity, and 76 normal weight)	Brazil	Plasma selenium concentration was negatively associated with obesity and visceral adiposity. Women with obesity reduced plasma and erythrocyte and increased urinary excretion of selenium.
Fontanelle et al., 2020 [83]	69 euthyroid women (35 with obesity and 34 normal weight)	Brazil	Negative association between plasma selenium and obesity, with higher clearance through excretion but not higher selenium levels in the urine.
Jardim-Botelho et al., 2016 [84]	153 infants aged 2–11 months	Brazil	91% of subjects with selenium deficiency
Retondario et al., 2019 [85]	76,957 adolescents aged from 12–17 years (49.7% girls)	Brazil	Prevalence of adequate selenium intake
Falcão et al., 2019 [86]	444 adolescents	Brazil	Intake of ultra-processed foods was correlated with inadequate selenium intake
Lane et al., 2021 [87]	891,723 subjects	-	Meta-analysis showed consumption of ultra-processed food was associated with increased risk of overweight
Stenzen et al., 2018 [88]	60 adolescents with severe obesity	Brazil	36% of selenium-deficient subjects based on serum selenium levels
Da Cunha et al., 2003 [89]	30 healthy adults	Brazil	Prevalence of mild selenium deficiency, particularly among men. No association between selenium and obesity

## Data Availability

Not applicable.
